# Improved visualization of peripherally inserted central catheters on chest radiographs of neonates using fractional multiscale image processing

**DOI:** 10.1186/s12880-018-0302-4

**Published:** 2019-01-07

**Authors:** Rebecca A. Hammon, Hannes Seuss, Matthias Hammon, Christian Grillhösl, Rafael Heiss, Martin Zeilinger, Nadine Bayerl, Pieter Vuylsteke, Friedrich Wanninger, Michael Schroth, Michael Uder, Oliver Rompel

**Affiliations:** 1 Department of Radiology, University Hospital Erlangen, Friedrich-Alexander-Universität (FAU) Erlangen-Nürnberg, Maximiliansplatz 3, 91054 Erlangen, Germany; 2 Department of Neonatology and Pediatric Intensive Care, Cnopf Children’s Hospital, Sankt-Johannis-Mühlgasse 19, 90419 Nürnberg, Germany; 3 Agfa HealthCare NV, Septestraat 27, 2640 Mortsel, Belgium

**Keywords:** Chest radiograph, Catheter tip visualization, Diagnostic confidence, PICC, Neonates, Post-processing, Fractional multiscale image processing

## Abstract

**Background:**

Peripherally inserted central catheters (PICCs) provide secure intravenous access for the delivery of life-sustaining medications and nutrition. They are commonly used in pediatrics. Confirmation of correct central catheter tip position is crucial. Verification is usually done by a radiograph. The aim of this study is to evaluate the ability of Fractional Multiscale image Processing (FMP) to detect PICC tips on the digital chest radiographs of neonates.

**Methods:**

A total of 94 radiographs of 47 patients were included in the study. 29 patients were male, 18 were female. The mean age of all examined children was 9.2 days (range 0–99 days). In total, six readers (two radiologists, two residents in radiology, one last year medical student, one neonatologist) evaluated 94 unprocessed and catheter-enhanced radiographs using a 5-point Likert scale (1 = poor catheter tip visualization, 5 = excellent catheter tip visualization). Additionally, the two radiologists evaluated the diagnostic confidence for chest pathologies using a 5-point Likert scale (1 = poor diagnostic confidence, 5 = excellent diagnostic confidence). Radiographs were evaluated on a dedicated workstation.

**Results:**

In all cases, the catheter-enhanced radiograph rated higher than (*n* = 471), or equal (*n* = 93) to, the unprocessed radiograph when visualizing catheter tips. 87% of the catheter-enhanced radiographs obtained a rating of 4 or higher, while only 42% of unprocessed radiographs received 4 or more points. Regarding diagnostic confidence for chest pathologies one radiologist rated two catheter-enhanced radiographs higher than the unprocessed radiographs, while all other 186 evaluations rated the catheter-enhanced radiographs equal to (*n* = 78) or lower than (*n* = 108) the unprocessed radiographs. Only 60% of the catheter-enhanced radiographs yielded a diagnostic confidence of 4 or higher, while 90% of the unprocessed images received 4 or more points.

**Conclusion:**

Catheter-enhanced digital chest radiographs demonstrate improved visualization of low contrast PICC tips in neonates compared to unprocessed radiographs. Furthermore, they enable detection of accompanying chest pathologies. However, definitive diagnosis of chest pathologies should be made on unprocessed radiographs.

## Background

Peripherally inserted central catheters (PICCs) provide secure intravenous access to deliver life-sustaining medications and nutrition. They are commonly used in pediatrics. The small luminal diameter is necessary for small neonates. PICCs can be inserted from the patient’s bedside and can remain in place for several weeks [1]. They are defined as central if their tips reside in the superior vena cava (SVC), right atrial junction, high inferior vena cava (IVC) or above the level of the diaphragm for lower extremity catheters [2]. Some authors include the subclavian veins as central veins too; however, central access is sometimes difficult to obtain, with success rates in pediatric patients being lower than in adults [3, 4]. This is especially true for premature neonates [5].

Complications of PICCs in neonates and children include infection, accidental dislodgement, occlusion, local infiltration, breakage and thrombosis [2, 6–8]. Previous studies have identified risk factors including young age, severity of illness, catheter dwell time, insertion site and catheter tip location [9, 10]. PICCs with a non-central catheter tip location are especially known to have an increased rate of complications [2, 11, 12].

Therefore, confirmation of central catheter tip position immediately after insertion or even during the placement procedure is crucial. Verification is usually done by a radiograph. While maintaining a sterile field, immediate reposition of the catheter, if necessary, is possible at this stage [1]. Fast visualization of small catheters’ tip on direct radiography (DR) or computed radiography (CR) chest radiographs can sometimes be difficult. Parameters are set to primarily produce the best average image to demonstrate a wide range of pulmonary, mediastinal and other abnormalities. Consequently, fine translucent catheters can easily be masked [13].

In adults, automatic processing algorithms which enhance radiographs and emphasize the edges of devices are able to improve visualization of central catheters on digital chest radiographs [13, 14].

The aim of this study is to evaluate the effects of fractional multiscale image processing (FMP) on enhanced detectability of PICC tips on DR chest radiographs of neonates.

## Methods

The institutional Ethics Committee of the University Hospital Erlangen/Germany approved the study. All procedures performed in studies involving human participants were in accordance with the ethical standards of the institutional research committee and with the 1964 Helsinki declaration and its later amendments or comparable ethical standards. The need for informed consent was waived by the Ethics Committee.

### Patient characteristics

A total of 94 radiographs of 47 patients were included in the study. 29 patients were male, 18 were female. The mean age of the patients at the day of the first examination was 8.8 ± 17.7 days (range 0 to 99 days). The mean age of the patients (for all examinations) was 9.2 ± 14.2 days (range 0–99 days). The average number of examinations per patient was 2.0 ± 1.6 (range 1–8). At the time of the first examination, the patients had a mean weight of 1920 ± 1040 g (range 480–4340 g), a mean height of 42 ± 7.2 cm (range 30–57 cm), a body mass index (BMI) of 9.7 ± 2.4 kg/m^2^ (range 5.3–15.0 kg/m^2^) and a mean body surface area (BSA) of 0.15 ± 0.05 m^2^ (range 0.06–0.26 m^2^). The body surface area was calculated with a formula proposed by Mosteller: body surface area (in m^2^) equals the square root of height (in cm) times weight (in kg), all divided by 3600. Patient demographics and diagnoses are provided in Tables 1 and 2.

**Table 1:** Patients’ demographics. The body surface area (BSA) was calculated with a formula proposed by Mosteller: body surface area (in m^2^) equals the square root of height (in cm) times weight (in kg), all divided by 3600. BMI = body mass index

	Minimum	5th percentile	Median	Mean	95th percentile	Maximum
Age (days)	0	0	2	8.8	32	99
Weight (g)	480	550	1770	1919	3900	4340
Height (cm)	30	30	43	42	54	57
BMI (kg/m^2^)	5.3	6.1	9.5	9.7	13.9	15.0
BSA (m^2^)	0.06	0.07	0.15	0.15	0.25	0.26

**Table 2:** Interpretation of chest radiographs: Chest pathologies. Of the 94 image pairs analyzed, 13 were without pathological findings. 81 cases demonstrated one or more chest abnormalities. For each image pair with abnormal findings, one main chest pathology was determined

Diagnosis	Number
Normal radiograph	13
Pneumonia	27
Infant respiratory distress syndrome (IRDS)	22
Bronchopulmonary dysplasia	9
Left-to-right shunting with pulmonary hypervolemia	8
Transient tachypnea of the newborn (TTN)	5
Pulmonary interstitial emphysema	5
Pneumothorax	4
Pleural effusion	1

### Radiographs

Digital radiographs were acquired with a dedicated system (DX-D 100 mobile, Agfa, Mortsel, Belgium) equipped with the Multiscale Image Contrast Amplification (MUSICA®) acquisition workstation with an integrated 17-in. touch-screen console. A digital wireless detector panel was used (DX-D 35C, 35 × 27.4 cm effective image size, 38.4 × 30.7 cm detector housing size, Agfa, Mortsel, Belgium).

### Catheters

Vygon Premicath micro-catheters were used (size: 28G, 0.17 × 0.35 mm; single-lumen; length: 20 and 30 cm; Vygon GmbH & Co.KG, Aachen, Germany).

### Post-processing software

The standard image processing and the dedicated processing for catheter enhancement are both based on Agfa’s MUSICA® image processing technology. The Multi-Scale Processing decomposes the image into several layers, each representing local contrast at a specific scale and adjusts these multiscale layers individually and automatically. The adjusted layers are finally reassembled into an image for display with increased visibility of details. Due to the way MUSICA® processes the various levels of contrast and detail in the radiograph, all the details in the image are displayed simultaneously without creating distracting and unwanted noise in the image. With the optional catheter processing a second companion image is created that focuses attention on tiny tubular structures, aiding the radiologist at identifying and visually following the catheter outline down to the tip. Catheter processing is based on multiscale image processing called FMP. Conventional multiscale decomposition is computed using a series of spatial filters. Typically, these filters compute a weighted average of pixels in a local neighborhood surrounding each pixel in the image, called the filter kernel. On the other hand, the filtered kernels are decomposed into smaller fractions at each scale in FMP; thus, the individual kernel fractions are enhanced instead of the weighted sum. A dedicated MUSICA® version, based on FMP technology, was developed with specific adjustments to the catheter enhancement. This algorithm extension achieved detailed contrast along the edges without introducing unwanted artifacts and with appropriate control of noise.

### Evaluation

In total, 6 readers (2 radiologists, 1 neonatologist, 2 residents in radiology, 1 last year medical student) evaluated all 94 unprocessed and catheter-enhanced radiographs using a 5-point Likert scale (1 = poor catheter tip visualization, 5 = excellent catheter tip visualization). Catheter tip locations are provided in Table 3. Additionally, the 2 radiologists evaluated the diagnostic confidence for chest pathologies using a 5-point Likert scale (1 = poor diagnostic confidence, 5 = excellent diagnostic confidence). The two reads were performed on pseudonymized studies, in random order, and at least two weeks apart. Radiographs were evaluated on a dedicated workstation with a 30-in. screen with a resolution of 3280 × 2048 pixels at 127.32 ppi (Coronis Fusion 6MP LED (MDCC-6230), Barco, Kortrijk, Belgium).

**Table 3:** Location of peripherally inserted central catheter (PICC) tips. Location of PICC tips in absolute values and percentage

Location	Number
Inferior vena cava	23 (25%)
Superior vena cava	21 (22%)
Right atrium	21 (22%)
Right subclavian vein	14 (16%)
Left subclavian vein	4 (4%)
Left brachiocephalic vein	4 (4%)
Left jugular vein	3 (3%)
Right jugular vein	2 (2%)
Left pulmonary artery	1 (1%)
Right ventricle	1 (1%)

### Statistical analysis

Quantitative variables are expressed as a mean ± SD and range; whereas, categorical variables are expressed as frequencies or percentages. The ratings were analyzed for independence using Pearson’s chi-squared test. For the correlation between the patients’ biometrical data and the Likert-Score, the Spearman’s rank correlation coefficient was used. SPSS 21 (IBM Corporation, Armonk, NY, USA) was used for the statistical analysis. A *p*-value < 0.05 was considered statistically significant.

## Results

### Catheter tip visualization

**Fig. 1:**
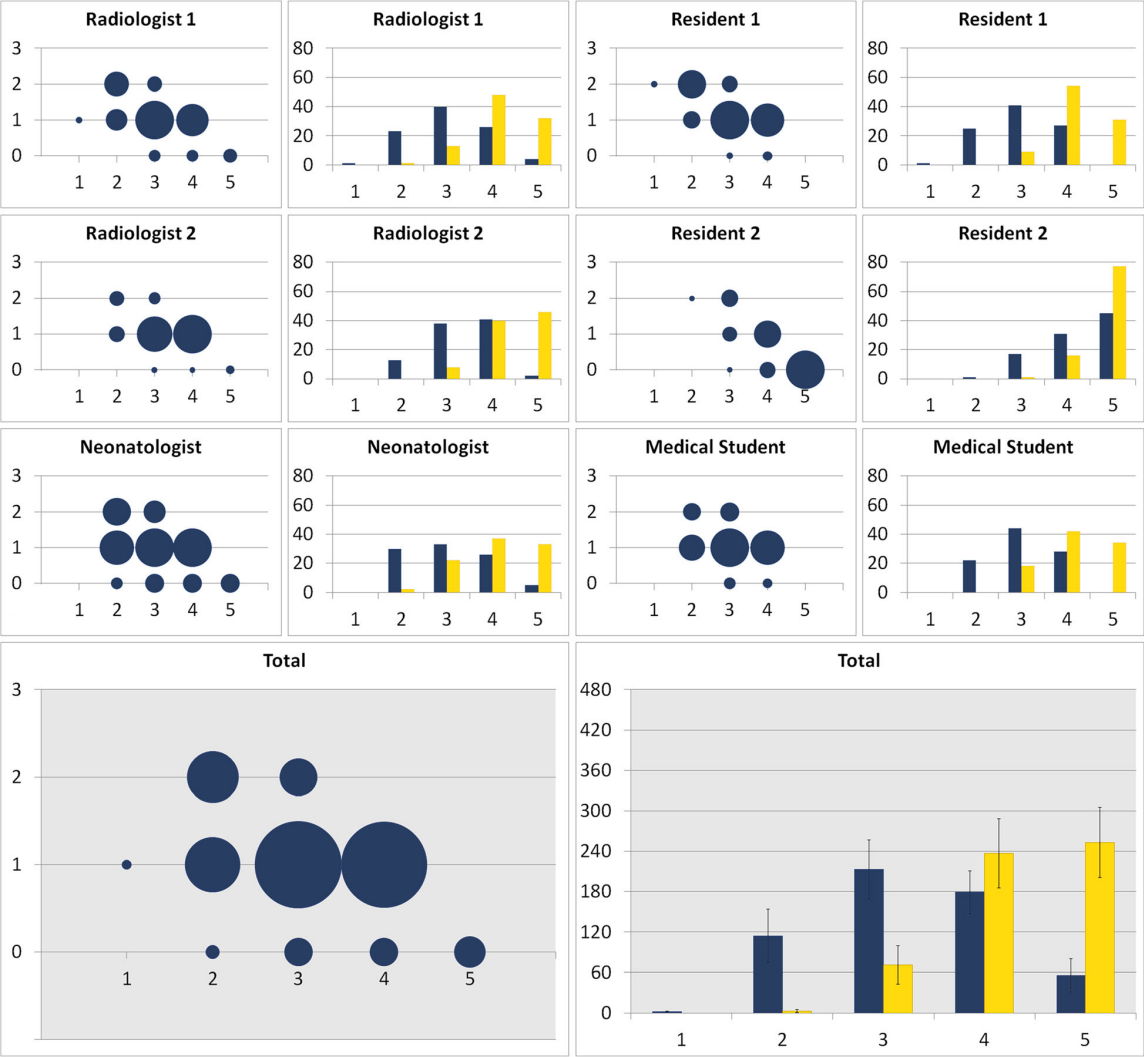
Catheter tip visualization. Bubble Chart: X-axis: Likert-score (1 = poor catheter tip visualization, 5 = excellent catheter tip visualization) of the unprocessed radiograph. Y-axis: Likert-score improvement of the catheter-enhanced radiograph; e.g. 1 = the catheter-enhanced radiograph score is one point higher than the unprocessed radiograph score. The size of each bubble indicates the number of scores. Bar Graph: Number of scores in each Likert-category; blue = unprocessed radiographs,  yellow = catheter-enhanced radiographs. In all cases the catheter-enhanced radiographs were rated equal or superior to the unprocessed radiographs

All six raters evaluated all 94 examinations (*n* = 564) regarding the visibility of the tip of the PICC on a Likert-scale from 1 (poor) to 5 (excellent). The tip of the PICC was visible in all unprocessed and catheter-enhanced images. In all cases, the catheter-enhanced image was rated higher than (*n* = 471) or equal to (*n* = 93) the unprocessed image. 87% of the processed images obtained a rating of 4 or higher, while only 42% of unprocessed images received 4 or more points. For all six raters, the distribution of the Likert-Score differed significantly between the catheter-enhanced and the unprocessed images (*p* < 0.001). The Likert score is loosely anti-correlated to the weight (r_s_ = − 0.212), height (r_s_ = − 0.229), BMI (r_s_ = − 0.188) and BSA (r_s_ = − 0.223) of the patient (p < 0.001). There is no significant correlation with patients age (r_s_ = − 0.024; *p* = 0.429). Detailed information is provided in Fig. 1 and Table 4.

**Table 4:** Catheter tip visualization and diagnostic confidence for chest pathologies. Six readers (2 radiologists, 1 neonatologist, 2 residents in radiology, and 1 last year medical student) evaluated 94 unprocessed and catheter-enhanced radiographs using a 5-point Likert scale (1 = poor catheter tip visualization, 5 = excellent catheter tip visualization). The 2 radiologists evaluated the diagnostic confidence for chest pathologies using a 5-point Likert scale (1 = poor diagnostic confidence, 5 = excellent diagnostic confidence)

Likert-Score	Catheter visualization		Diagnostic confidence for chest pathologies	
	Unprocessed	Catheter enhanced	Unprocessed	Catheter enhanced
1	2	0	0	2
2	114	3	4	15
3	213	71	14	58
4	179	237	102	82
5	56	253	68	31

### Diagnostic confidence for chest pathologies

**Fig. 2:**
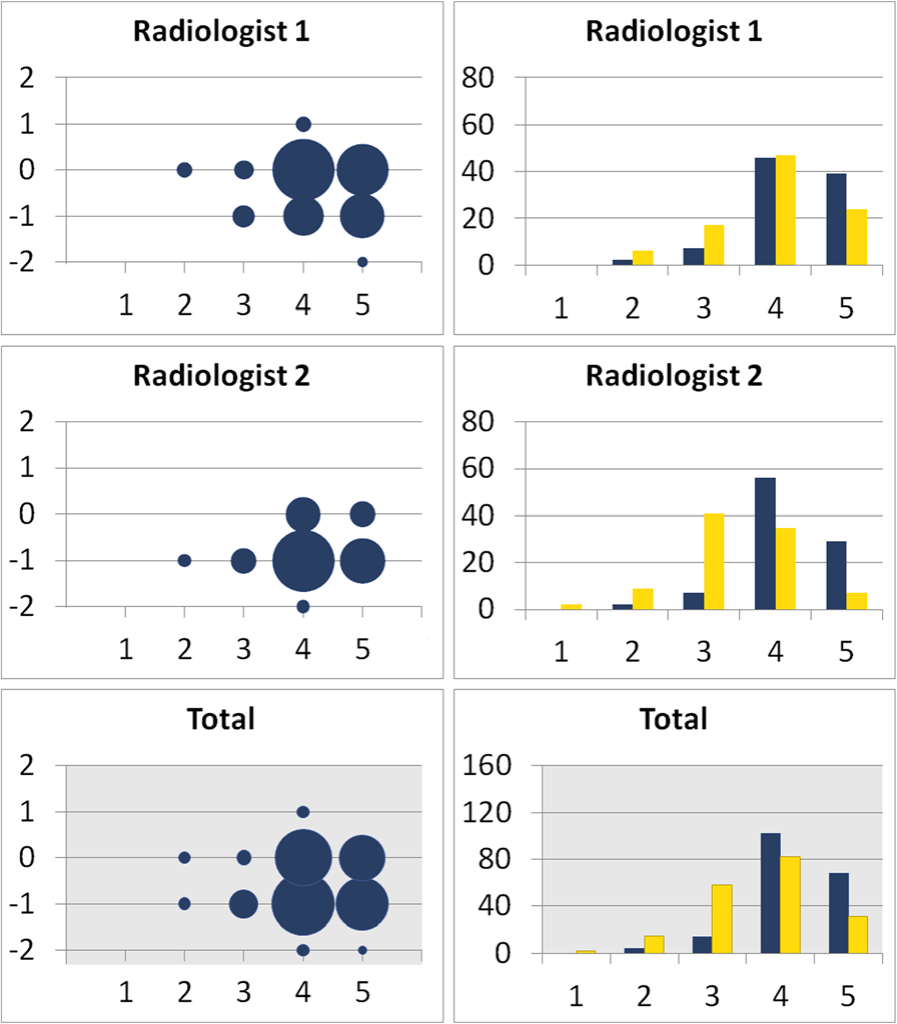
Diagnostic confidence for chest pathologies. Bubble Chart: X-axis: Likert-score (1 = poor catheter tip visualization, 5 = excellent catheter tip visualization) of the unprocessed radiograph. Y-axis: Likert-score improvement of the catheter-enhanced radiograph; e.g. -1 = the catheter-enhanced radiograph score is one point lower than the unprocessed radiograph score. The size of each bubble indicates the number of scores. Bar Graph: Number of scores in each Likert-category; blue = unprocessed radiographs, yellow = catheter-enhanced radiographs. Radiologist 1 reports a better diagnostic confidence for chest pathologies in two catheter-enhanced radiographs. In all other cases the unprocessed radiographs were rated equal or superior to the catheter-enhanced radiographs

**Fig. 3:**
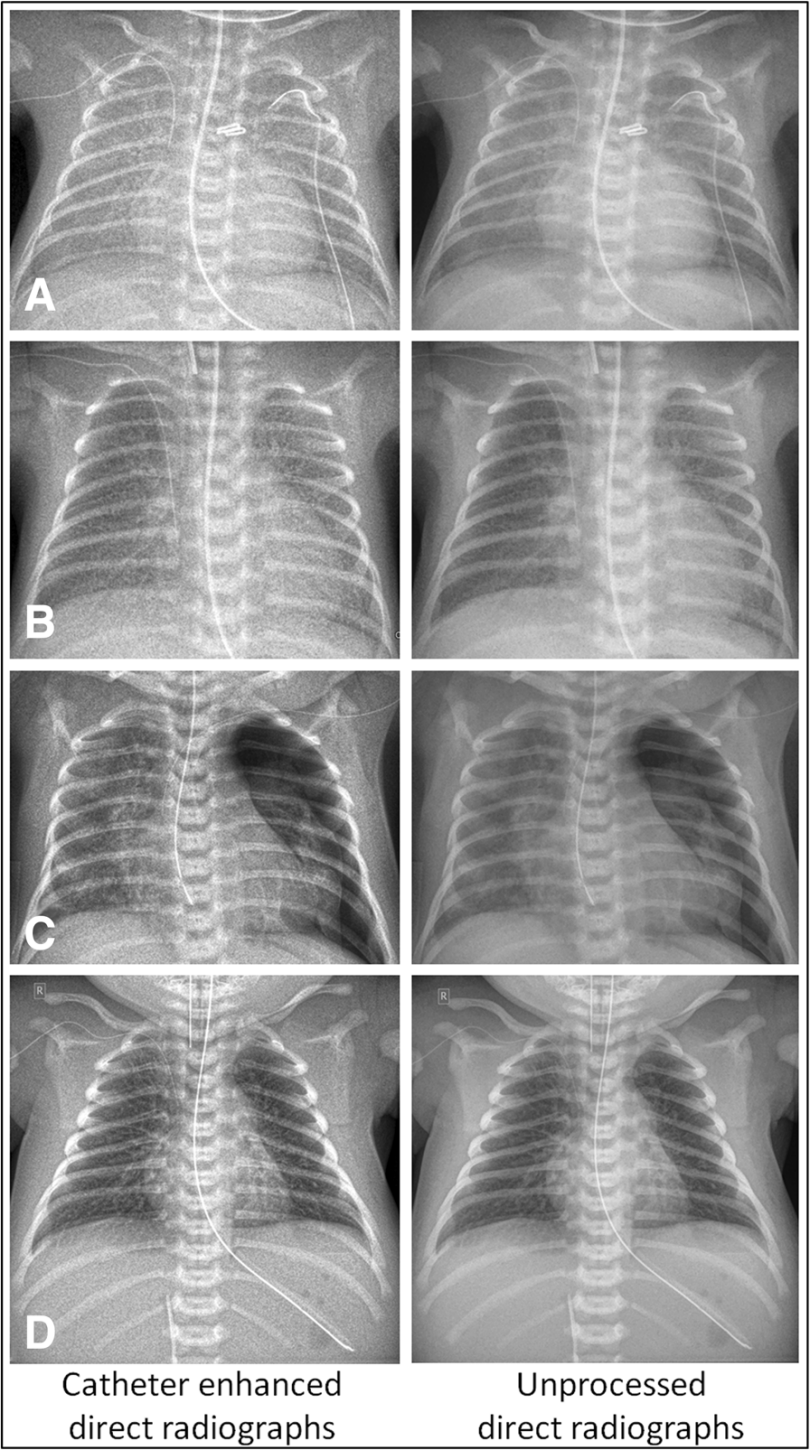
Exemplary comparisons of catheter-enhanced and unprocessed direct radiographs. **a** A central catheter is inserted in a vein of the right upper limb and ends in the superior vena cava. Main diagnosis: Left-to-right shunting with pulmonary hypervolemia, shortly after surgical ligation of a patent ductus arteriosus (PDA). **b** A central catheter is inserted in a vein of the right upper limb and ends in the right atrium. Main diagnosis: Infant respiratory distress syndrome (IRDS). **c** A central catheter is inserted in a vein of the left upper limb and ends in the superior vena cava. Main diagnosis: Two-sided pneumonia, left-sided pneumothorax. **d** A central catheter is inserted in a vein of the right upper limb and ends in the superior vena cava. Main diagnosis: Transient tachypnea of the newborn (TTN)

The two radiologists also rated their diagnostic confidence for chest pathologies on the Likert-scale (*n* = 188). One radiologist rated two catheter-enhanced images higher than the unprocessed images. In all other 186 evaluations, the processed images were rated equal to (*n* = 78) or lower than (*n* = 108) the Likert scale ratings for unprocessed images. Only 60% of the catheter-enhanced images yielded a diagnostic confidence of 4 or higher, while 90% of unprocessed images received 4 or more points. The distribution of ratings differed significantly between both groups (p < 0.001 for *Radiologist 1*; *p* = 0.021 for *Radiologist 2*). The Likert-Score is not significantly correlated to the weight (r_s_ = − 0.012), height (r_s_ = − 0.026), BMI (r_s_ = 0.035), BSA (r_s_ = − 0.014) and age (r_s_ = − 0.011) of the patient (*p* > 0.5). Detailed information is provided in Fig. 2 and Table 4. Exemplary comparisons of catheter-enhanced and unprocessed direct radiographs are shown in Fig. 3.

## Discussion

This study assessed the impact of FMP on the detectability of PICC tips in the digital chest radiographs of neonates. We show that catheter-enhanced images significantly improve the visualization of the fine line tips when compared to unprocessed radiographs. Moreover, we found that catheter-enhanced images enable detection of important accompanying pathologies, although the diagnostic confidence for chest pathologies had a lower rating than the unprocessed images.

In the literature, the rate of adverse events that precipitate PICC removal in pediatric patients range from 17 to 50% [8, 10, 15]. Nevertheless, the association between catheter tip location and PICC complications is controversial. Thiagarajan et al. found that PICCs placed in non-central veins provide safe and reliable intravenous access [4]; while alternative studies were able to prove that PICCs terminating in non-central venous locations have higher complication risks [11, 12, 16]. In the study of Jumani et al., PICCs with a non-central tip location were more than three times as likely to be removed after an adverse event, when compared to PICCs with a central tip location. There was a strong association with non-infectious complications [2]. A catheter tip lying in a non-central location may touch the vessel wall more often, which can irritate and disrupt the endothelial cell layer and trigger coagulation. At worst, vascular erosions may lead to life-threatening complications. Neonates and small children are at highest risk because of structurally smaller and less stable vessels [2, 7, 11, 17]. No significant difference between complication rates of upper and lower extremity insertion sites of non-centralized PICC tip location was found [18].

In a study by Fricke et al., PICC placement without imaging guidance resulted in incorrect non-central tip position in 86% of the case studies, which required catheter reposition [19]. Increasing evidence supports the use of ECG confirmation of central catheter placement in adults, but evidence is limited in neonates [20].

Fast and secure verification of adequate central catheter tip position in neonates is a worthwhile goal. Optimally, PICC line tips should be located in the SVC or IVC, close to the junction with the right atrium, 0.5–1 cm outside of the cardiac chambers in premature infants and 1–2 cm in larger infants, respectively [21].

Johnson et al. evaluate the use of ultrasound-guided PICC placement as an effective technique with excellent success rates in very low birth weight infants [5]. Consequently, the number of manipulations decreases and the frequency of control radiographs can be reduced with this method [22–24]. In a study by Jain et al., targeted neonatal echocardiography (TNE) was evaluated for assessing correct central position of PICC line tips. The results show that TNE is superior to plain radiographs. The sensitivity of radiographs in determining malposition was only 64% with a specificity of 55%, while echocardiography proved to be more helpful in real-time line manipulation post-PICC insertion [25]. Although ultrasonography is obviously attractive, substantial training is required for its appropriate use and for the interpretation of results [20].

PICC placement with fluoroscopic guidance can be a safe and successful alternative, but cannot be done from the patient’s bedside [19, 21].

Digital chest radiography remains the mainstay for confirming proper catheter position since it simultaneously enables reliable detection of various intrathoracic pathologies. DR offers better image quality than CR for bedside chest radiography [26].

Chest radiographs taken on intensive care units are often suboptimal due to incorrect positioning in seriously sick patients. Multiple devices, overlying leads and wires, and dressings or sheets can obscure detection of fine tubular structures [27]. Additionally, catheter tips can be easily obscured by the brighter regions of the image, such as the heart and mediastinum, or may be superposed by bones or complex anatomical structures [13].

DR radiographs are usually viewed on digital workstations, allowing gray-scale windowing to increase contrast in brighter regions. Thus, the PICC visualization can be improved [13]. On the other hand, a dedicated catheter processing software such as FMP eliminates time-consuming manual re-processing, because it is tuned to automatically enhance the visibility of the fine, translucent catheter material. Similarly, images enhanced with FMP significantly facilitate the assessment of correct catheter tip position without the need for further manual adjustments. Once verification is complete, a bedside screen mounted on a mobile radiographic system may be used to immediately reposition the catheter, if necessary.

A limitation of the study is that the radiographs were not evaluated on the bedside screen of the mobile radiographic system. This would be of interest and clinically relevant because PICC radiographs are likely to be evaluated at bedside.

## Conclusions

FMP enables catheter enhancement in chest radiographs, which allows improved visualization of low contrast PICC tips in neonates compared to unprocessed radiographs. Catheter-enhanced images are particularly helpful for instant assessment of correct tip placement. Furthermore, they enable detection of accompanying pathologies. However, a definite diagnosis of chest pathologies should be made on unprocessed radiographs.
